# Survival Extrapolation Incorporating General Population Mortality Using Excess Hazard and Cure Models: A Tutorial

**DOI:** 10.1177/0272989X231184247

**Published:** 2023-07-13

**Authors:** Michael J. Sweeting, Mark J. Rutherford, Dan Jackson, Sangyu Lee, Nicholas R. Latimer, Robert Hettle, Paul C. Lambert

**Affiliations:** Statistical Innovation, AstraZeneca, Cambridge, UK; Department of Population Health Sciences, University of Leicester, UK; Statistical Innovation, AstraZeneca, Cambridge, UK; Department of Population Health Sciences, University of Leicester, UK; School of Health and Related Research, University of Sheffield, Sheffield, UK; Delta Hat Limited, UK; Health Economics and Payer Evidence, AstraZeneca, Cambridge, UK; Department of Population Health Sciences, University of Leicester, UK; Department of Medical Epidemiology and Biostatistics, Karolinska Institutet, Sweden

**Keywords:** survival extrapolation, health technology assessment, excess hazard models, modeling, overall survival

## Abstract

**Background:**

Different parametric survival models can lead to widely discordant extrapolations and decision uncertainty in cost-effectiveness analyses. The use of excess hazard (EH) methods, which incorporate general population mortality data, has the potential to reduce model uncertainty. This review highlights key practical considerations of EH methods for estimating long-term survival.

**Methods:**

Demonstration of methods used a case study of 686 patients from the German Breast Cancer Study Group, followed for a maximum of 7.3 y and divided into low (1/2) and high (3) grade cancers. Seven standard parametric survival models were fit to each group separately. The same 7 distributions were then used in an EH framework, which incorporated general population mortality rates, and fitted both with and without a cure parameter. Survival extrapolations, restricted mean survival time (RMST), and difference in RMST between high and low grades were compared up to 30 years along with Akaike information criterion goodness-of-fit and cure fraction estimates. The sensitivity of the EH models to lifetable misspecification was investigated.

**Results:**

In our case study, variability in survival extrapolations was extensive across the standard models, with 30-y RMST ranging from 7.5 to 14.3 y. Incorporation of general population mortality rates using EH cure methods substantially reduced model uncertainty, whereas EH models without cure had less of an effect. Long-term treatment effects approached the null for most models but at varying rates. Lifetable misspecification had minimal effect on RMST differences.

**Conclusions:**

EH methods may be useful for survival extrapolation, and in cancer, EHs may decrease over time and be easier to extrapolate than all-cause hazards. EH cure models may be helpful when cure is plausible and likely to result in less extrapolation variability.

**Highlights:**

- In health economic modeling, to help anchor long-term survival extrapolation, it has been recommended that survival models incorporate background mortality rates using excess hazard (EH) methods.
- We present a thorough description of EH methods with and without the assumption of cure and demonstrate user-friendly software to aid researchers wishing to use these methods.
- EH models are applied to a case study, and we demonstrate that EHs are easier to extrapolate and that the use of the EH cure model, when cure is plausible, can reduce extrapolation variability.
- EH methods are relatively robust to lifetable misspecification.

Estimates of long-term survival are frequently required in cost-effectiveness analyses of new treatments.^1,2^ Such analyses play an important role in reimbursement decisions for new interventions and rely on estimates of lifetime benefits and costs. In oncology, the limited follow-up of clinical trials usually necessitates extrapolation of survival beyond the trial period.^3^ However, cost-effectiveness estimates can be highly sensitive to the extrapolation method used.^4^ A review of National Institute for Health and Care Excellence (NICE) cancer Technology Appraisals has identified a variety of extrapolation approaches that have previously been used, often employing parametric survival models.^5^ To help avoid extremely implausible projections, it has been recommended that standard parametric models incorporate background mortality rates and/or other relevant external information.^6^

Incorporation of general population mortality (GPM) rates into survival extrapolation has the potential to prevent implausible projections by using GPM rates as an anchor for long-term hazards. Previously, Technology Appraisals have used GPMs outside of the model-fitting process to switch from the parametric model projections to the GPM rates when the projected rates hit the GPM rates.^7,8^ This causes a discontinuity in the all-cause hazard function at the time the parametric rates drop below the GPM rates. Furthermore, parametric models may project mortality rates that remain implausibly higher than GPM rates into the future. A more statistically coherent approach to incorporating GPM rates directly into the modeling process is to use an excess hazard (EH) model.^9,10^ A EH model will ensure that all-cause hazards are at least as large as the GPM rates and may be larger if the model estimates nonzero excess mortality. The EH approach therefore deals with one of the issues with survival extrapolation, namely, preventing projected mortality rates dropping below GPM rates. The EH approach partitions overall mortality rates into expected rates determined from the GPM rates and excess rates, estimated from the model, which describe the additional hazard experienced in the study population. The GPM rates are assumed fixed and known and are usually taken from population lifetables, which are matched to the study population by age, sex, and calendar year. Once an EH model has been fitted, predictions of excess mortality can be combined again with GPM rates to give estimates of long-term all-cause survival. This approach may be appealing given patterns of excess mortality rates, and GPM rates are likely to be very different over time.^6^

As an example, Figure 1 shows the background GPM hazard (red line) and the EH (blue line) in one arm of a hypothesized randomised controlled trial (RCT). The all-cause hazard (green line) is the sum of the GPM and EHs. Due to trial inclusion/exclusion criteria, the all-cause hazard rate may start out low before increasing and then decreasing as the EH decreases. In the long term, the all-cause hazard may start increasing again as it starts to become dominated by the background GPM rate (red line), which increases as the cohort ages. Therefore, in this example, the all-cause hazard function has 2 turning points (at 3 and 11 y), whereas the EH function has just 1 turning point (at 3 y) and so has a less complex shape.

**Figure 1:**
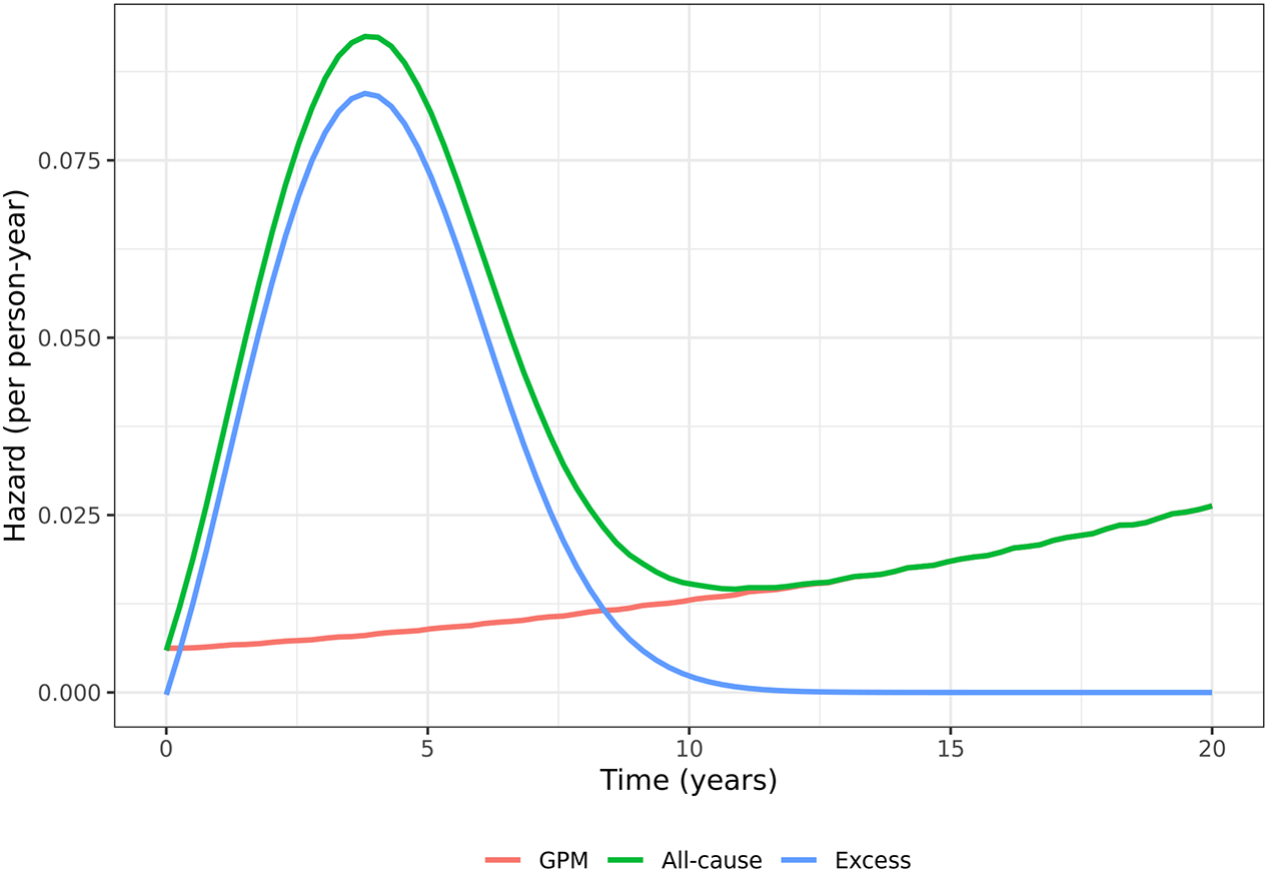
Hypothesised hazard functions in a cancer clinical trial where cure is possible.

A class of models called EH cure models consider the possibility of cure, which can lead to the excess mortality rates approaching zero over time. If the assumption of cure is considered reasonable, the use of cure models may be appealing and provide improved extrapolations.^11^

The focus of this article is on the demonstration of the use of EH methods for extrapolation using a suite of standard parametric models that have been recommended for consideration in health economic modeling.^2^ We demonstrate the methods using data from the German Breast Cancer Study Group (GBCS), which is freely available and for which R code is provided in Appendix 3 and R and Stata code is online at https://github.com/AstraZeneca/survextrap-excesshazards. Although we make no firm recommendations on the use of EH methods for survival extrapolation, this tutorial provides a hands-on demonstration for researchers wishing to use EH methods and discusses the underlying assumptions, robustness of the estimation to lifetable misspecification, and how predictions of all-cause survival, hazard, and restricted mean survival time (RMST) can be obtained. Finally, we consider the plausibility of extrapolated long-term treatment effects using fully stratified parametric models that do not rely on strong proportional hazards (PH) or constant acceleration factor (AF) assumptions.

## Methods

### EH/Relative Survival Framework

In an EH framework, the all-cause mortality rate for an individual i in the study population, $h_{i}\left(t\right)$ , is broken into 2 constituent parts, the background mortality rate $h_{i}^{*}\left(t\right)$ and the excess mortality rate $\lambda_{i}\left(t\right)$ , such that, based on an additive hazards assumption,

$$h_{i}\left(t\right)\;=\;h_{i}^{*}\left(t\right)\;+\lambda_{i}\left(t\right).$$

In an EH model, the background mortality rate is treated like an offset and is assumed fixed and known. It is individual specific as it is usually based on a lifetable matched by variables such as age, sex, and calendar year.

Using the transformation between the hazard and survival scale, the all-cause survival $S_{i}\left(t\right)$ is a product of the background (expected) survival $S_{i}^{*}\left(t\right)$ and the relative survival, $R_{i}\left(t\right)$ :

$$S_{i}\left(t\right)\;=\;S_{i}^{*}\left(t\right)R_{i}\left(t\right).$$

$R_{i}\left(t\right)$ is known as the relative survival function as it describes the ratio of all-cause survival to background survival.

GPM rates are commonly used for the background mortality, whereas a parametric survival distribution can be chosen to model the excess rate. A model that includes covariates, for example, treatment, could then be defined using a proportional (excess) hazards (PH) model

$$\lambda_{i}\left(t\right)=\lambda_{0}\left(t\right)\exp\left(\beta^{T}\;X_{i}\right)$$

or an accelerated failure time (AFT) model such that on the relative survival scale

$$R_{i}\left(t\right)=\;R_{0}\left(t\exp\left(-\beta^{T}\;X_{i}\right)\right).$$

Alternatively, parametric models could be fitted to each treatment arm separately. This approach relaxes the PH/AFT assumption and places fewer constraints on how the treatment effect varies over time.

In the short time frame of a typical RCT, the excess mortality rate is often not too dissimilar to the all-cause rate, as many deaths will be associated with the disease under study. However, in the long term, the excess and all-cause rates will start to diverge. In oncology, excess rates will tend to decrease and may even approach zero if cure is possible (see hypothesized example in Figure 1).

### EH Cure Models

EH cure models take into account the possibility of cure, which can lead to the EH tending toward zero over time, which is not guaranteed for all EH models. There are 2 types of cure model: the mixture-cure and non–mixture-cure model.^12^ Both have a long history, and their properties have been studied widely.^13–15^ In this tutorial, we consider only the mixture-cure model, which expresses the relative survival as a mixture of 2 latent subpopulations, one that is cured and never experiences mortality due to the disease and an uncured subpopulation. The all-cause survival probability for an individual i is written as

(1) $$S_{i}\left(t\right)\;=\;S_{i}^{*}\left(t\right)R_{i}\left(t\right)\;=\;S_{i}^{*}\left(t\right)\left(\pi_{i}\;+\;\left(1-\pi_{i}\right)S_{u,i}\left(t\right)\right)$$

where $\pi_{i}$ is the probability that the individual will be cured of their disease and $S_{u,i}\left(t\right)$ is a parametric survival function for the uncured component of the mixture. The EH cure model is expressed in the framework of an EH model but with $R_{i}\left(t\right)$ modeled as a mixture. If π is not a function of covariates, then it can be interpreted as an overall cure fraction (the proportion of the population estimated to be eventually cured of their disease if other causes of mortality were not acting on the population). However, research has shown that the cure fraction should be interpreted cautiously since it may be very sensitive to model misspecification^14^ and can be unstable.^15^ In the special case where π=0 , mixture-cure models collapse to standard EH models. For the purposes of extrapolation in this tutorial, we consider the incorporation of GPM rates for all applications of the cure model.

### Obtaining Predictions from EH Models

EH models estimate parameters on the EH scale, including EH ratios. To get predictions of all-cause survival and all-cause hazard, we need to reincorporate the GPM rates. The predicted all-cause survival for an individual i at time t is the predicted relative survival (obtained from the EH model) multiplied by their expected survival at time t :

(2) $${\hat{S}}_{i}\left(t\right)=\;S_{i}^{*}\left(t\right){\hat{R}}_{i}\left(t\right).$$

The prediction of all-cause survival is individual specific even if no covariates are included in the EH model, because the expected mortality rates will typically vary by the age, sex, and calendar year of the individual. Usually, interest is in the marginal predicted all-cause survival. This is the survival distribution for the trial (or trial arm) population, which is calculated via averaging (standardizing) individual-level survival curves over a suitable target population (e.g., the original study population). Given N individuals in the target population, the marginal all-cause survival at time t is predicted as

(3) $$\bar{S}\left(t\right)\;=\sum_{i=1}^{N}\;\frac{{\hat{S}}_{i}\left(t\right)}{N}$$

The hazard function for the marginal all-cause survival at time t is a weighted average of the N individual all-cause hazard functions, weighted by the probability of survival by time t :

(4) $$\bar{h}\left(t\right)\;=\;\frac{\sum_{i=1}^{N}{\hat{S}}_{i}\left(t\right){\hat{h}}_{i}\left(t\right)\;}{\sum_{i=1}^{N}{\hat{S}}_{i}\left(t\right)\;}$$

where ${\hat{h}}_{i}\left(t\right)=h_{i}^{*}\left(t\right)+{\hat{\lambda }}_{i}\left(t\right)$ is the predicted all-cause hazard for individual i . When covariates are used in the EH model, the same calculations can be performed, resulting in marginal estimates of the all-cause survival and hazard function, averaging over the covariate distribution of the N individuals. If an indicator of treatment (or exposure), Z , is included in the EH model, then a counterfactual marginal contrast can be obtained, in which a marginal estimate is calculated assuming all patients had received the treatment ( Z=1 ) and contrasted against a marginal estimate assuming all patients did not receive the treatment ( Z=0 ).

### Software Implementation

All models were fitted using the flexsurv and flexsurvcure packages in R. Postestimation predictions of all-cause survival, hazard, RMST, and EHs were calculated using the standsurv function within flexsurv, which calculates marginal survival and hazard measures set out in equation 3 and equation 4. Standard errors and confidence intervals for these marginal effects are calculated using the delta method. To calculate implied hazard ratios and differences in RMST from models fitted separately to each treatment (or exposure) group, we fit fully stratified survival models where treatment (or exposure) is included as a covariate that affects all parameters in the model (e.g., both the shape and scale parameters for a standard Weibull, or the mean, standard deviation and cure parameters in a log-normal cure model). Fully stratified survival models are equivalent to fitting models to each treatment arm separately but have the additional advantage of allowing contrasts and standard errors of contrasts between treatment groups to be easily calculated using existing software implementation. Example code used to fit these models and to produce the predictions are given in Appendix 3, with full code online at https://github.com/AstraZeneca/survextrap-excesshazards.

## Results

The German Breast Cancer Study (GBCS) group provides data on 686 primary node-positive breast cancer patients diagnosed between 1984 and 1989. The median age at diagnosis is 53 y (Q1–Q3; 46–61 y). The data contain information on survival and recurrence times together with their respective censoring indicators. The data also contain a variety of patient characteristics, including age and diagnosis date. For this demonstration, cancer grade is used as a prognostic (exposure) variable, collapsed into 2 levels: grades 1/2 and grade 3. The objective is to obtain extrapolated survival curves up to 30 y after diagnosis and to compare the 2 prognostic groups. Cancer grade was used for illustrative purposes because it produced 2 distinct survival curves. There were 171 deaths with a mean follow-up to death or censoring of 3.6 y (2,480 person-years of follow-up) and maximum follow-up of 7.3 y. A Kaplan-Meier plot of the data, stratified by grade, is shown in Supplementary Figure 1. At 6-y follow-up, survival was estimated to be 66% and 48% in the grade 1/2 and grade 3 groups, respectively.

### Model Fit for Cancer Grades 1 and 2

Seven standard parametric survival models were fitted to the group with cancer grades 1/2 (Exponential, Weibull, Log-logistic, Log-normal, Gompertz, Gamma, Generalised Gamma). The Exponential distribution makes a very strong assumption of a constant hazard over time. Three other distributions have monotonic hazards (Weibull, Gamma, Gompertz), whereas the remaining 3 distributions (Log-logistic, Log-normal, Generalized Gamma) allow unimodal hazard functions with a single turning point.

EH models with and without cure were fitted by incorporating background mortality rates from (West) German lifetables obtained from the Human Mortality Database (https://www.mortality.org). These were matched by age, sex, and calendar year to patients in the breast cancer study. The lifetables used in this example were from 1956 to 2020 and for ages 0 to 119 y. Predicted expected survival beyond the maximum age or calendar year in the lifetable used the rate at the maximum for as many years as required.

### AIC Statistics and Root Mean Squared Prediction Error

Goodness of fit of the 8 standard parametric models, as assessed via the Akaike information criterion (AIC) statistic, are shown in Table 1 (first column). The Log-normal with the lowest AIC gives the best fit, whereas the Exponential and Gompertz are shown to provide the worst fit among the models considered. The Generalized Gamma distribution is second best and has a similar AIC to the Log-normal.

**Table 1:** AIC Statistics for 7 Parametric Survival Models and the Extended Excess Hazard Models with and without Cure, Fitted to the Grade 1/2 Group of the GBCS Data Set^a^

Distribution	Standard Parametric	Excess Hazard (No Cure)	Excess Hazard (Cure)
Exponential	874.6 (7)	844.4 (7)	846.5 (7)
Weibull	843.2 (5)	815.7 (5)	812.3 (5)
Gompertz	858.1 (6)	829.3 (6)	825.7 (6)
Gamma	840.2 (3)	812.8 (3)	808.4 (3)
Log-logistic	840.3 (4)	813.2 (4)	809.6 (4)
Log-normal	835.4 (1)	806.6 (2)	804.9 (2)
Generalized Gamma	837.3 (2)	801.4 (1)	803.4 (1)

AIC, Akaike information criterion; GBCS, German Breast Cancer Study.

a Rank order statistics are shown in parentheses. Note that AIC statistics are not directly comparable between excess hazard and standard parametric models.

Most statistical software that fit EH models do not use the full likelihood, and as such, AIC statistics reported from EH models cannot be compared directly with AIC statistics reported from standard parametric models. Appendix 1 provides a more complete discussion of this issue. Nevertheless, AIC statistics can be used to compare between EH models with different distributions and between EH cure models. In the EH models without cure, the Generalized Gamma is the model that now gives the best AIC, whereas the Exponential and Gompertz continue to have a poor relative fit (Table 1, second column).

When a cure assumption is imposed, the goodness of fit improves for all parametric models, except the Exponential and Generalized Gamma, in comparison with the EH models without cure (Table 1, third column).

AIC goodness-of-fit statistics, along with visual fit to the data, can be useful tools to rule out clearly ill-fitting models. However, we warn against selecting a single model based on AIC alone, as similar-fitting models can lead to very different extrapolations.

A further approach to understanding model fit is to compare root mean squared prediction error (RMSPE) between predicted marginal all-cause survival and the Kaplan-Meier estimator. This metric allows direct assessment of the marginal fit of the model and has the additional benefit of allowing us to compare between different EH and non-EH models. Further details of the RMSPE is given in Appendix 2. The RMSPE statistics show improved fit using EH models for some parametric distributions and worse fit for others (Supplementary Table 1).

### All-Cause Hazard Plots

All-cause hazard functions for the 3 approaches are shown in Supplementary Figure 2, with a B-spline smoothed empirical hazard function.^16^ The observed hazard increases over the first 2 y, which then plateaus and slightly rises again between years 5 and 6. Many of the standard parametric and EH models without cure have an increasing hazard that does not capture the plateau. Only the Log-normal and Generalized Gamma models show a plateauing of the hazard function. The various EH cure models behave more similarly to each other, and all the models except for the Exponential now incorporate a turning point in the all-cause hazard function but appear to underestimate the all-cause hazard at 6 y.

### Cure Fraction Estimates

Estimated cure fractions are between 58% and 67% for 5 of the 7 EH cure models (Supplementary Table 2). For the Exponential (the worst-fitting model) and the Generalized Gamma (the best-fitting model), the estimated cure fraction is 0%. The Generalized Gamma has more flexibility to model the EH function for the uncured subpopulation and estimates a low (but nonzero) EH in the long term for the uncured component. This effectively mimics cure and discounts the need for a cure component in the model.

### Extrapolated Survival and Hazard for Cancer Grades 1 and 2

**Figure 2:**
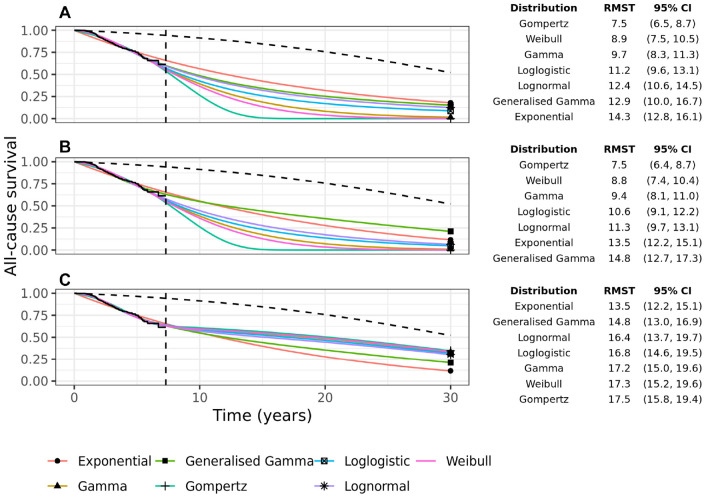
All-cause survival extrapolation of 7 parametric models for grades 1/2 breast cancer patients: (a) without external data, (b) with background mortality rates incorporated using an excess hazards model, and (c) with background mortality rates incorporated in a mixture-cure excess hazards model. The black dashed line shows the marginal background survival. The vertical dashed line shows the end-of-study follow-up. The table shows the 30-y restricted mean survival times (RMST) with 95% confidence intervals.

All-cause survival extrapolations up to 30 y are extremely variable for the 7 parametric models without background mortality (Figure 2a). This is evidenced by the 30-y RMST estimates, which range from 7.5 y under the Gompertz model to 14.3 y under the Exponential model. This variability remains when using EH models without cure (Figure 2b) but decreases when using EH cure models (Figure 2c). The 30-y RMST under the cure models ranges from 13.5 y to 17.5 y.

**Figure 3:**
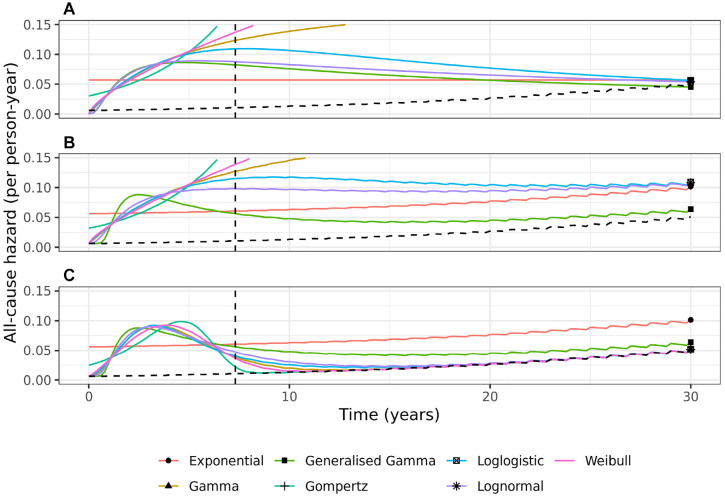
Extrapolation of all-cause hazards estimated from 7 parametric models for grades 1/2 breast cancer patients: (a) without external data, (b) with background mortality rates incorporated using an excess hazards model, and (c) with background mortality rates incorporated in a mixture-cure excess hazards model. The black dashed line shows the marginal background hazard. The vertical dashed line shows end of study follow-up.

To better understand each model, we need to study the all-cause hazard functions over a 30-y period, as plotted in Figure 3. The Weibull, Gompertz, and Gamma distributions have unrealistic increasing hazards that go beyond the range of the *y*-axis when applied both without and with background mortality rates. The models might be discounted due to lack of face validity, since the hazards rise rapidly and are 2.9, 4.8, and 74.5 times the average rate (estimated by the Exponential model) by 20 y, for the Gamma, Weibull, and Gompertz standard parametric models, respectively. The Log-logistic, Log-normal, and Generalized Gamma standard parametric models all have a turning point and tend toward background mortality rates by 30-y, whereas the all-cause hazard function stays consistently above background rates when these models are applied in an EH (no cure) setting. For 5 of the EH cure models, the EH rate becomes negligibly small between 7 and 15 y, and hence, the all-cause hazard reaches and follows the background hazard. The plausibility of this assumption along with the credibility of the long-term all-cause survival predicted by the cure models should be carefully considered and justified if a cure model is to be used. The Exponential and Generalized Gamma EH cure models (the worst-fitting and best-fitting model, respectively) predict an all-cause hazard that remains above the background hazard up to 30 y.

### Long-term Effects of Cancer Grade

The model-fitting process is repeated using all patients in the GBCS data to investigate the long-term effect of cancer grades 1 and 2 versus grade 3, using cancer grade to mimic the 2 arms of an RCT. It is common in health technology assessment submissions to fit entirely separate survival models to 2 treatment arms of a clinical trial; this avoids assuming the treatment effect follows a PH or constant AF assumption, which may be unrealistic over a long time period. Conversely, however, the treatment effect is now unrestricted and governed entirely by the shape of the extrapolated hazards in the 2 arms. The implied long-term treatment effect of fitting 2 separate models to the treatment arms is often not fully investigated. We assume the same underlying distributional form for the 2 cancer grade groups; that is, if one group is fitted using a Weibull distribution, then the other group is also fitted using a Weibull. We return to this issue of using the same “type” of model in the discussion.

**Figure 4:**
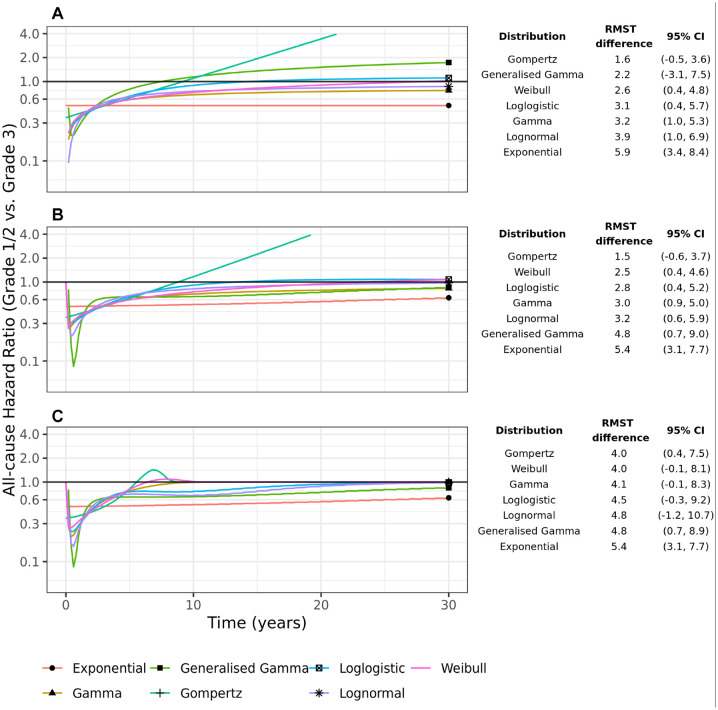
All-cause hazard ratios derived from 7 parametric models fitted separately to the 2 cancer grade groups: (a) without external data, (b) with background mortality rates incorporated using an excess hazards model, and (c) with background mortality rates incorporated in a mixture-cure excess hazards model. The table shows the difference in 30-y restricted mean survival times (RMST) with 95% confidence intervals.

The implied all-cause hazard ratio from fitting models to each group separately is shown in Figure 4. In the standard parametric models, there is considerable variability in the long-term all-cause hazard ratios with the Gompertz hazard ratio going above 1 after 10 y and continuing to rise thereafter. The variability in the hazard ratios is translated to large differences between the groups in RMST at 30 y, ranging from a difference of 1.6 y (95% CI −0.5, 3.6) for the Gompertz model to 5.9 y (95% CI 3.4, 8.4) for the Exponential.

The EH models reduce this variability somewhat. Most of the models predict an increasing all-cause hazard ratio that tends to and approaches 1. The variability in the RMST difference has reduced, although it is still considerable.

In the EH cure models, the all-cause hazards in the 2 groups tend toward background hazards, albeit at possibly different rates, and so the all-cause hazard ratio tends towards 1 in the long-term (Figure 4c). For most of the models, the all-cause hazard ratio reaches 1 between 10 and 25 y. The exception is the Exponential (a poor-fitting model) and the Generalized Gamma, where the all-cause hazard ratio gets close to but does not reach 1 by 30 y. The variability in the RMST difference has reduced considerably, with most models predicting a difference in RMST of between 4 and 4.8 y. However, the confidence intervals are quite variable between the models, with the 95% confidence intervals for the Log-normal being particularly wide.

Supplementary Figure 3 shows the all-cause hazard functions for the 2 groups for the Generalized Gamma and Log-normal distributions. The all-cause hazards for the EH models for the 2 groups have similar shapes and tend to converge after a period of time, tracking either above the GPM rates (EH noncure models) or converging towards the GPM rates (EH cure models).

### Sensitivity of Cure Models to Choice of External Population Hazards

This section highlights the robustness (or otherwise) of the EH models based on the choice of lifetable and how variation in background mortality rates between a selection of different countries affects estimation of EH rates and extrapolations. We consider 2 scenarios: first, where an incorrect lifetable is used in both the estimation of the EH model and in the prediction of all-cause survival, and second, where an incorrect lifetable is used in the estimation but a common lifetable is used for prediction. The latter scenario considers the same target estimand (country) but considers misspecification of the EHs estimated from the EH model.

GPMs were obtained from the Human Mortality Database^17^ for 6 countries (West Germany, Hungary, Chile, United States, Sweden, Japan). There is reasonable variation in the GPM rates from these countries (Supplementary Table 3). The Log-normal EH models with and without cure were refitted using background mortality rates from each of these 6 countries in turn. In scenario 1, predictions were then made for the country whose lifetable was used for estimation, whereas in scenario 2, predictions were made for a West German population (the target population) using the estimated EH models.

**Figure 5:**
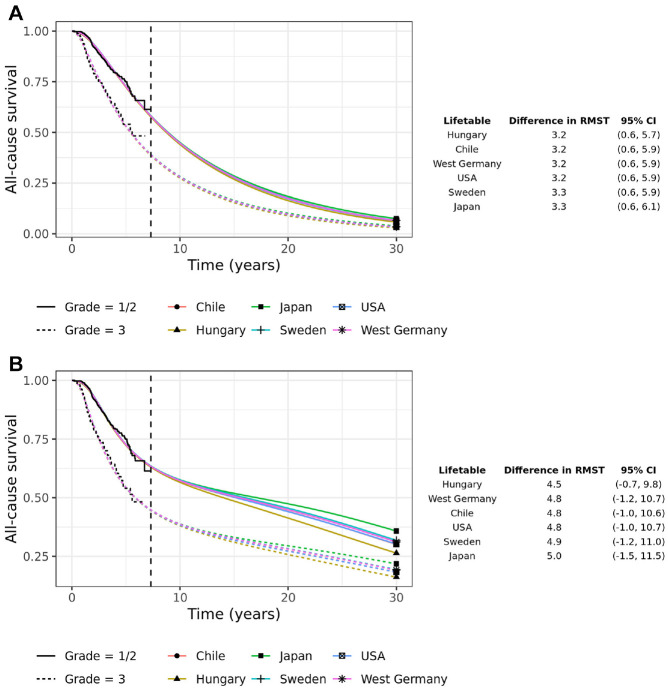
All-cause survival extrapolation of the stratified Log-normal excess hazard models with and without cure, where background mortality rates used for model fitting are taken from 1 of 6 countries and predictions are made for the country used in the estimation. The table shows the difference in 30-y restricted mean survival time (RMST) with 95% confidence intervals. (a) Scenario 1: Excess hazard model without cure (same lifetable for estimation and prediction), (b) Scenario 1: Excess hazard model with cure (same lifetable for estimation and prediction).

Changing the lifetable in the estimation of the EHs and then applying the same background rates to predict all-cause survival has very little effect on predictions if an EH model without cure is fitted (Figure 5a). However, using an EH cure model results in much wider variability, which is a consequence of different background rates dominating the long-term predictions (Figure 5b).

In scenario 2, there is very little variability in the predicted all-cause survival for a West German population using an EH noncure model dependent on the lifetable used in the estimation of the excess rates, whereas there is slightly more variability using an EH cure model (Supplementary Figure 4). The difference in RMST at 30 y is relatively robust to the choice of lifetable, although there are clear differences between the noncure and cure models.

In conclusion, based on these limited investigations, results are generally robust to lifetable misspecification. This finding is supported by previous research that investigated the use of different projected general population rates in calculating life expectancy in colon cancer patients.^18^

The implications of this finding are that it may be plausible to fit a single EH (cure) model utilising the lifetable information that best matches the study population. Health economic evaluations in different countries could then use these excess mortality/relative survival estimates and combine with background mortality rates/expected survival from the country of interest using equation 2 to predict all-cause survival in that jurisdiction. This approach makes the stronger assumption that excess mortality rates are commutable between countries whereas other-cause rates vary.

## Discussion

This tutorial demonstrates the potential that EH models with and without cure have for improving the practice of survival extrapolation in Technology Appraisals, when long-term extrapolation is required. EH cure models are likely to result in less extrapolation variability due to the use of GPM data and the cure assumption they make but should be considered only if cure is deemed plausible. If cure is not plausible, then cure models can result in large bias, as shown previously in simulation studies.^6^ In cancer studies, extrapolation based on the EH function is more stable and reliable, as it is likely to have a simpler long-term shape than the all-cause hazard and in cancer is likely to decrease over time.^19^ Hence, standard parametric functions with at most 1 turning point in the hazard function may be more suitable when applied to the EH compared with the all-cause hazard. Furthermore, EH cure models typically force a turning point in the all-cause hazard function as the hazard returns toward the background hazard in the long term, even if simpler monotonic hazard functions are used for the uncured subpopulation. The EH models use background mortality rates, which typically dominate the long-term extrapolation reflecting the aging cohort. None of the standard parametric models incorporate this external information. Although EH models were applied in this tutorial to a breast cancer population, the model framework can also be used for extrapolating survival in other cancers and to nonmalignant diseases in which GPM rates are relevant and a dominating factor for informing long-term rates.

Consideration should be given to whether GPM rates are suitable as long-term estimates of background mortality. It may be plausible that hazards remain above population mortality rates even in the long term, in which case background rates from other data sources (such as cancer registries with long-term follow-up) could be used within an EH modeling framework.

This tutorial does not discuss the use of flexible parametric models (FPMs) that use splines to model hazard functions since the purpose of the tutorial was to demonstrate EH methods using the standard suite of parametric models commonly used in Technology Appraisals. Nevertheless, FPMs are becoming increasingly popular as they can more accurately capture changes in the hazard function over follow-up and hence overcome some of the limitations of using standard parametric models.^6,19–21^ FPMs are more explicit in the assumption they make about the shape of the hazard after the end of follow-up. For example, the Royston-Parmar FPM on the log cumulative EH scale assumes linearity after the last knot,^20,22^ whereas an FPM cure model assumes zero EH after the last knot.^23^ The utility of these models may therefore lie in the ability to explicitly place the last knot at a location where these assumptions may be deemed reasonable. Meanwhile, the extrapolated (excess) hazard from standard parametric models is not made explicit in the model formulation, and we recommend plotting the long-term estimated (excess) hazard function to assess if it is deemed plausible.

Cure models are increasingly seen as an important tool for survival extrapolation. However, in some settings where cure is not reasonable, cure models can give very poor extrapolation performance.^6^ The parametric cure models discussed in this tutorial allow EH rates to approach (asymptote) zero over time, which in our application can be interpreted as patients eventually being free from breast cancer–related deaths. However, within the study follow-up, there may be no evidence of “cure.” Whether the assumption of long-term cure beyond the range of the data is reasonable or not is an untestable assumption (with the study data to hand) and so relies on arguments around biological plausibility, pharmacologic mechanisms, clinical opinion, and other external evidence.^21^ Care should be given not to overinterpret the estimated cure fraction in a model since EHs may tend to zero even when the cure fraction is zero. This was demonstrated in our case study, in which a Generalized Gamma EH model was flexible enough to capture a low long-term EH, negating the need for a mixture-cure model (and hence estimating a cure fraction of zero).

The choice of model used for extrapolation should not be based entirely on within-sample goodness-of-fit. As previously demonstrated,^11,24^ models with near-identical within-trial fit can provide qualitatively discrepant extrapolations. Therefore, within-trial goodness-of-fit should be used in conjunction with objective assessment of the credibility of extrapolations. This approach naturally leads to the consideration of external data sources such as other trials with longer-term follow-up, disease registries, and expert elicitation, and Bayesian approaches lend themselves naturally to the formal incorporation of external information.^25–27^

In the evaluation of relative treatment efficacy, a fully stratified parametric model does not rely on strong PH or constant AF assumptions. However, this approach does assume the same “type” of model is applied to both arms (e.g., a Weibull), as recommended in the NICE Technical Support Document 14.^2^ A yet more assumption-free approach could be to allow the parametric distribution as well as the parameters of the model to vary by treatment group. This may provide a wider range of possible treatment effects and is an avenue for further exploration.

In conclusion, this tutorial has demonstrated how EH methods can help reduce model variability through the incorporation of population mortality rates and can make explicit assumptions regarding long-term cure. We suggest these methods should be considered when conducting extrapolation of all-cause survival.

## Supplemental Material

sj-docx-1-mdm-10.1177_0272989X231184247 – Supplemental material for Survival Extrapolation Incorporating General Population Mortality Using Excess Hazard and Cure Models: A Tutorial Supplemental material, sj-docx-1-mdm-10.1177_0272989X231184247 for Survival Extrapolation Incorporating General Population Mortality Using Excess Hazard and Cure Models: A Tutorial by Michael J. Sweeting, Mark J. Rutherford, Dan Jackson, Sangyu Lee, Nicholas R. Latimer, Robert Hettle and Paul C. Lambert in Medical Decision Making
