# Supplementary material for: Survival Extrapolation Incorporating General Population Mortality Using Excess Hazard and Cure Models: A Tutorial
Source: Med Decis Making. 2023 Jul 13;43(6):737–48. doi: 10.1177/0272989X231184247 (PMC10422853; doi:10.1177/0272989X231184247)

**Supplementary Material for**

**Survival extrapolation incorporating general population mortality using excess hazard and cure models: a tutorial**

Contents

[Supplementary Figure 1. Kaplan-Meier survival plot of GBCS data stratified by cancer grade. 2](#_Toc125728268)

[Supplementary Figure 2. All-cause hazard functions of seven standard parametric models for Grades 1/2 breast cancer patients, a) without external data, b) with background mortality rates incorporated using an excess hazards model, and c) with background mortality rates incorporated in a mixture-cure excess hazards model. 3](#_Toc125728269)

[Supplementary Figure 3. All-cause hazards predicted for the Generalised Gamma and Log-Normal distributions when fitted separately to the two cancer grade groups, a) without external data, b) with background mortality rates incorporated using an excess hazards model, and c) with background mortality rates incorporated in a mixture-cure excess hazards model. 4](#_Toc125728270)

[Supplementary Figure 4. All-cause survival extrapolation of the stratified Log-Normal EH models with and without cure where background mortality rates used for model fitting are taken from one of six countries and predictions are made for the target West Germany population. The table shows the difference in 30-year restricted mean survival time (RMST) with 95% confidence intervals. 5](#_Toc125728271)

[Supplementary Table 1. Root mean squared prediction error for 7 parametric survival models and the extended excess hazard models with and without cure, fitted to the Grade 1/2 group of the GBCS dataset. Rank order statistics are shown in parentheses. 6](#_Toc125728272)

[Supplementary Table 2. Estimated cure fractions, rounded to 2 decimal places, with Wald 95% confidence intervals in the German Breast Cancer dataset from fitting seven parametric mixture-cure models. 7](#_Toc125728273)

[Supplementary Table 3. Mortality ratios comparing age, sex, and calendar year matched lifetables from five countries (USA, Hungary, Chile, Sweden, Japan) to West Germany. 8](#_Toc125728274)

[Appendix 1. AIC calculations in the excess hazards framework 9](#_Toc125728275)

[Appendix 2. Root mean-squared prediction error to assess marginal fit 11](#_Toc125728276)

[Appendix 3. Example R code: gbcsCS dataset 12](#_Toc125728277)

## Supplementary Figure 1. Kaplan-Meier survival plot of GBCS data stratified by cancer grade.


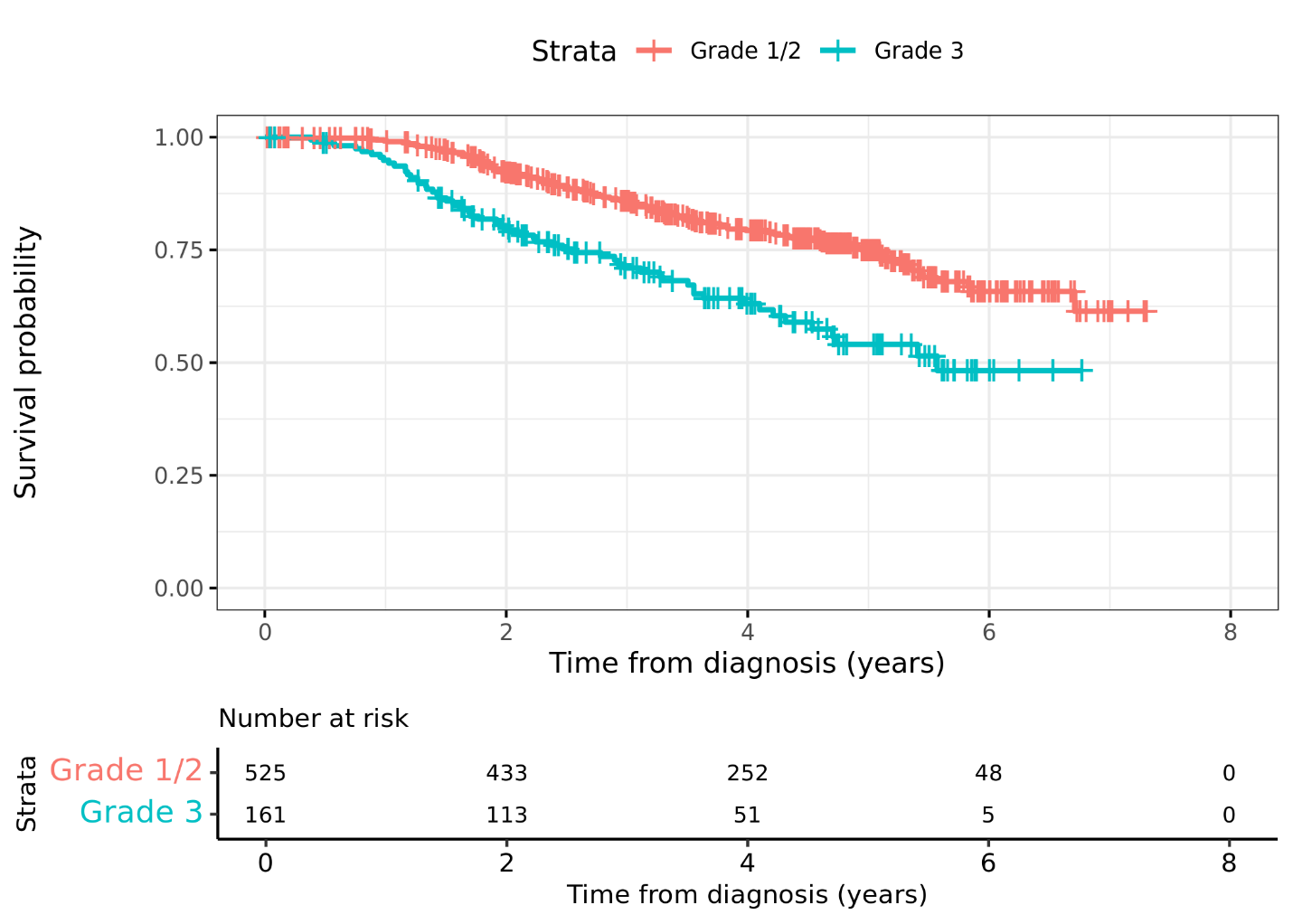


## Supplementary Figure 2. All-cause hazard functions of seven standard parametric models for Grades 1/2 breast cancer patients, a) without external data, b) with background mortality rates incorporated using an excess hazards model, and c) with background mortality rates incorporated in a mixture-cure excess hazards model.


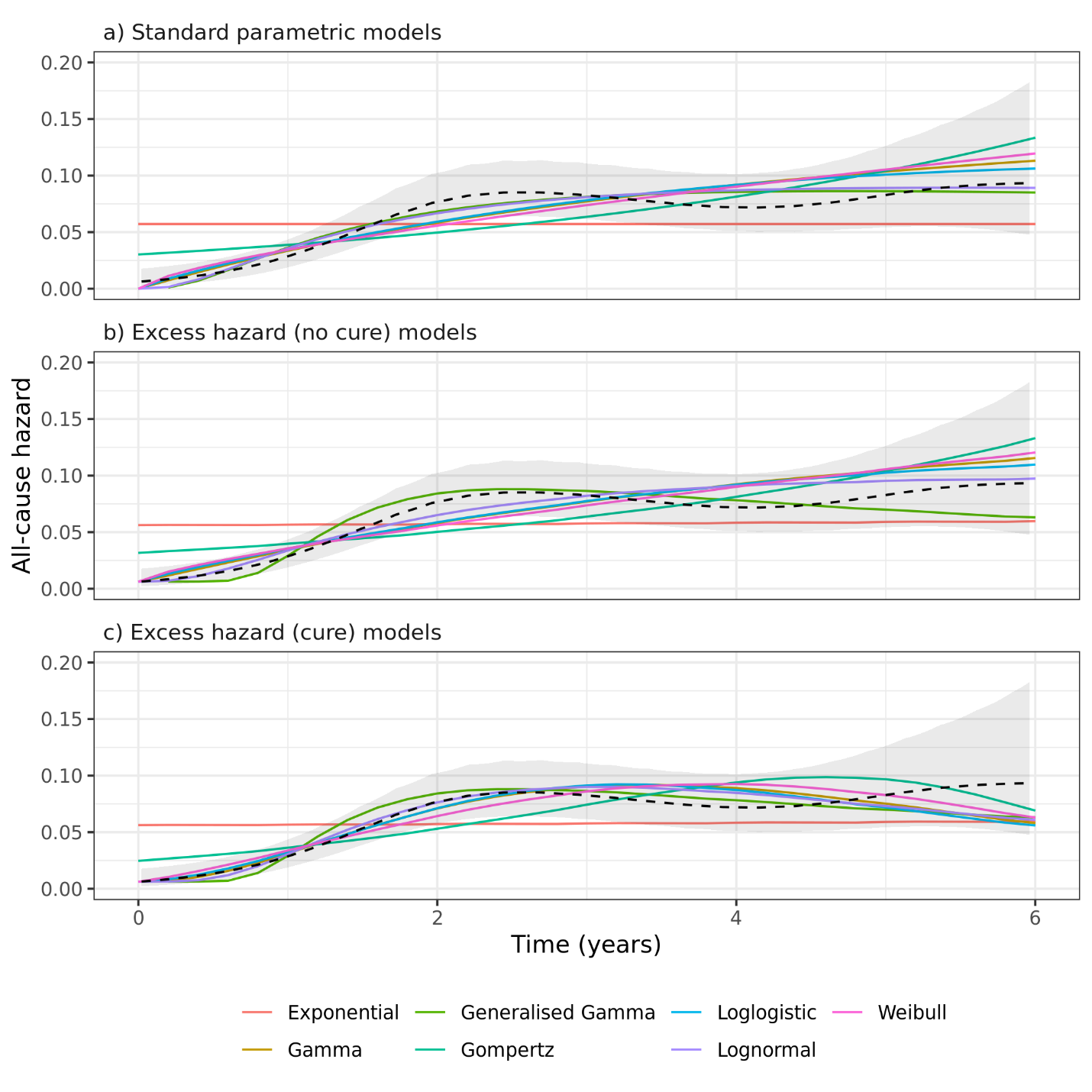
B-spline smoothed empirical hazards with 95% confidence intervals are shown as a black dashed line and shaded region, respectively.

## Supplementary Figure 3. All-cause hazards predicted for the Generalised Gamma and Log-Normal distributions when fitted separately to the two cancer grade groups, a) without external data, b) with background mortality rates incorporated using an excess hazards model, and c) with background mortality rates incorporated in a mixture-cure excess hazards model.


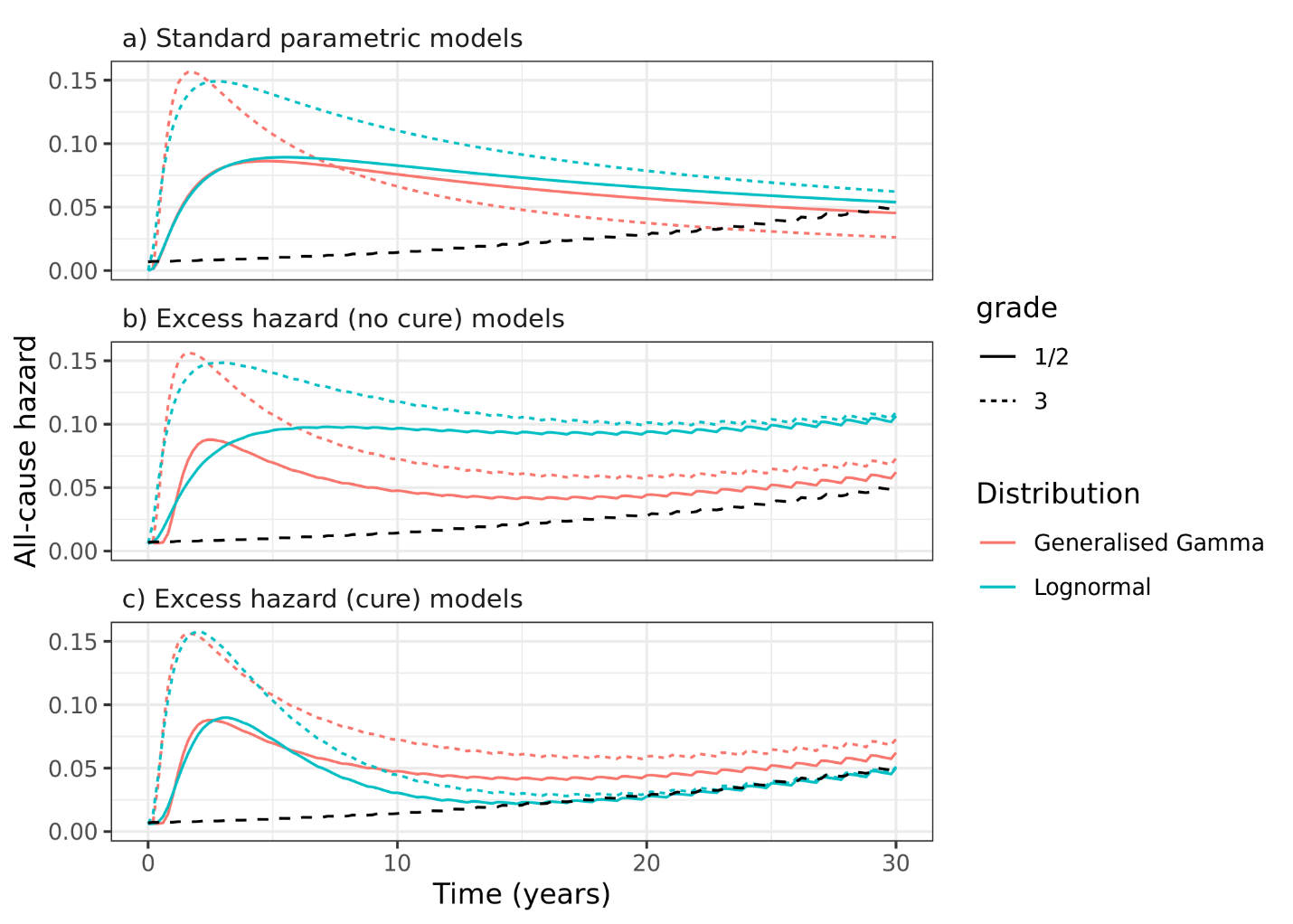


## Supplementary Figure 4. All-cause survival extrapolation of the stratified Log-Normal EH models with and without cure where background mortality rates used for model fitting are taken from one of six countries and predictions are made for the target West Germany population. The table shows the difference in 30-year restricted mean survival time (RMST) with 95% confidence intervals.


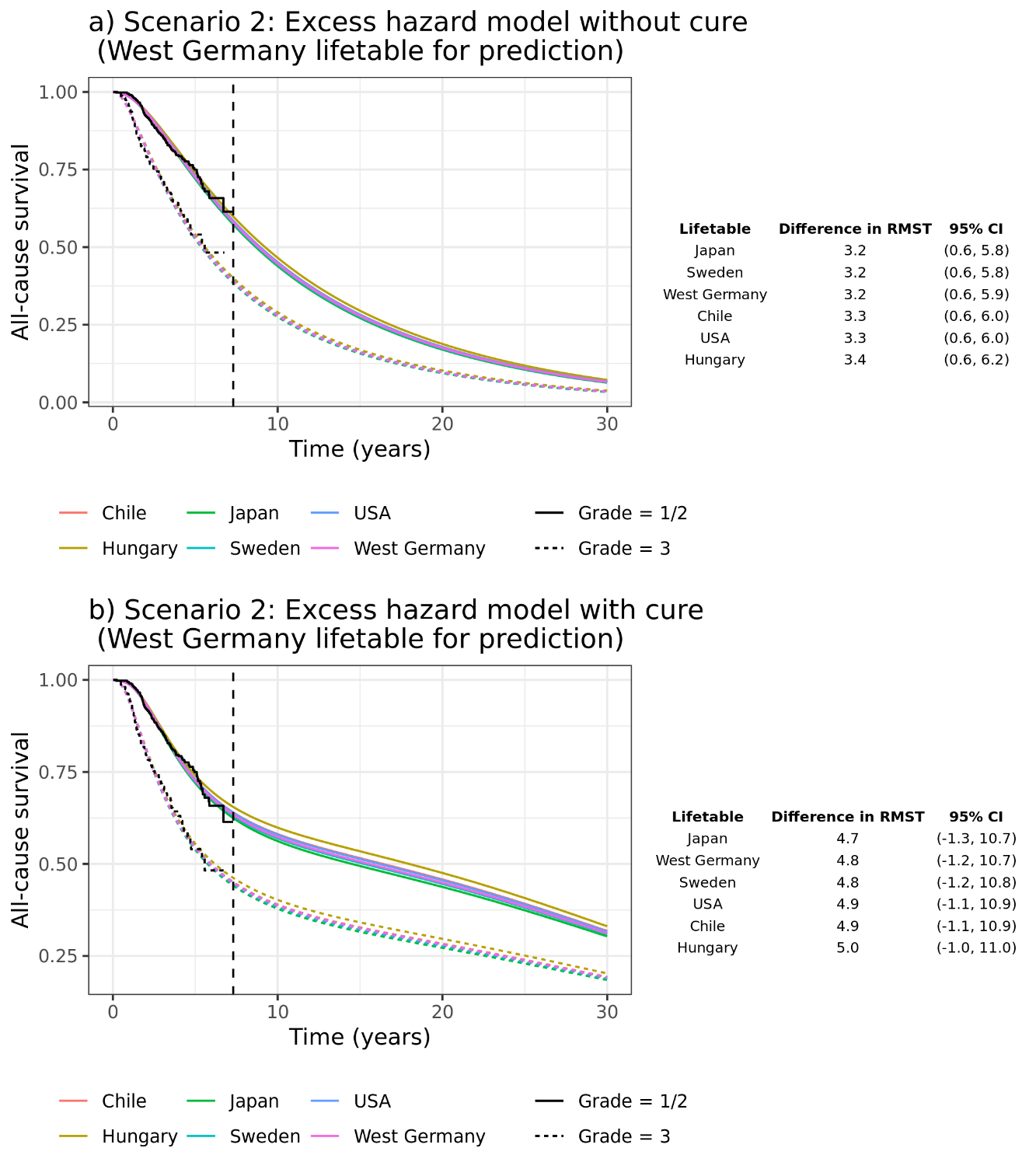


## Supplementary Table 1. Root mean squared prediction error for 7 parametric survival models and the extended excess hazard models with and without cure, fitted to the Grade 1/2 group of the GBCS dataset. Rank order statistics are shown in parentheses.

| Distribution | Standard parametric | Excess hazard (no cure) | Excess hazard (cure) |
| --- | --- | --- | --- |
| Exponential | 0.0287 (7) | 0.0276 (7) | 0.0276 (7) |
| Weibull | 0.0171 (5) | 0.0169 (4) | 0.0143 (3) |
| Gompertz | 0.018 (6) | 0.0177 (6) | 0.0145 (4) |
| Gamma | 0.0166 (4) | 0.0169 (5) | 0.0148 (5) |
| Log-logistic | 0.0154 (3) | 0.0159 (3) | 0.0152 (6) |
| Log-normal | 0.0119 (2) | 0.0141 (2) | 0.0141 (2) |
| Generalised Gamma | 0.0113 (1) | 0.0135 (1) | 0.0135 (1) |

## Supplementary Table 2. Estimated cure fractions, rounded to 2 decimal places, with Wald 95% confidence intervals in the German Breast Cancer dataset from fitting seven parametric mixture-cure models.

| Distribution | Cure fraction | AIC |
| --- | --- | --- |
| Exponential | 0 (0, 1) | 846.5 |
| Weibull | 0.65 (0.52, 0.76) | 812.3 |
| Gompertz | 0.67 (0.56, 0.75) | 825.7 |
| Gamma | 0.64 (0.5, 0.76) | 808.4 |
| Log-logistic | 0.61 (0.45, 0.75) | 809.6 |
| Log-normal | 0.58 (0.37, 0.77) | 804.9 |
| Generalised Gamma | 0 (0, 1) | 803.4 |

## Supplementary Table 3. Mortality ratios comparing age, sex, and calendar year matched lifetables from five countries (USA, Hungary, Chile, Sweden, Japan) to West Germany.

| Country | Mortality Ratio (median [Q1, Q3]) |
| --- | --- |
| West Germany | 1 |
| USA | 1.08 [0.87, 1.32] |
| Hungary | 1.20 [1.03, 1.62] |
| Chile | 1.16 [0.99, 1.52] |
| Sweden | 0.85 [0.74, 0.95] |
| Japan | 0.86 [0.75, 1.00] |

## **Appendix 1**. AIC calculations in the excess hazards framework

For individual $i$, let $t_{i}$ denote the observed survival time (time to event or censoring) and $d_{i}$ the event indicator, and $Y_{i} = (t_{i}, d_{i})$. For a standard parametric survival model with parameter vector $\theta$, the log-likelihood contribution for individual $i$ is

$\log L_{i}(\theta; Y_{i}) = d_{i}\log(h\left( t_{i} \right|\theta))+\log(S\left( t_{i} \right|\theta))$,

where $h(t)$ and $S(t)$ are the hazard and survival functions for the chosen parametric model, respectively.

Under an excess hazard / relative survival model with parameter vector $\theta_{EH}$ the log-likelihood contribution for individual $i$ can be written as

$\log L_{i}(\theta_{EH}; Y_{i}) = d_{i}\log\left( h_{i}^{*}\left( t_{i} \right)+\lambda\left( t_{i} \right|\theta_{EH}) \right) +\log\left( S_{i}^{*}\left( t_{i} \right) \right) +\log\left( R\left( t_{i} \right|\theta_{EH} \right))$,

where $\lambda\left( t \right|\theta_{EH})$ is the excess hazard function, $R\left( t \right|\theta_{EH}) =\exp\left( -\int_{0}^{t} \lambda\left( u \right|\theta_{EH}) du \right)$ is the relative survival function, and $h^{*}(t)$ and $S^{*}\left( t \right)=\exp\left( -\int_{0}^{t} h^{*}\left( u \right)du \right) =\exp\left( -H^{*}\left( t \right) \right)$ are the expected hazard and expected survival functions, respectively.

Since $\log\left( S_{i}^{*}\left( t_{i} \right) \right) = - H_{i}^{*}(t_{i})$ does not depend on model parameters it can be excluded from the likelihood. The log-likelihood and AIC statistics reported in statistical software such as flexsurv in R and stpm2 in Stata is therefore a reduced log-likelihood with the terms $\log\left( S_{i}^{*}\left( t_{i} \right) \right)$ missing as these terms are not needed for estimation. Direct comparisons with the log-likelihood / AIC statistics from standard parametric models is therefore not possible.

It is feasible that an estimate of $\log\left( S_{i}^{*}\left( t_{i} \right) \right) = -H_{i}^{*}(t_{i})$ could be reintroduced to each log-likelihood contribution if the individual cumulative expected hazards can be computed (e.g. from a lifetable). Such calculations are possible, for example in the survival package in R with the use of the survexp function and argument method = "individual.h".

It should be noted that, once the AIC statistic has been corrected, an EH model without covariates can be anticipated to give a better AIC statistic than its standard parametric counterpart (without covariates). This is because, although covariates are not incorporated into the excess hazard function, patient characteristics such as age and gender are used in lifetables to explain differential expected hazards. An implication of this observation is that age-specific all-cause survival can be anticipated to be better predicted by an EH model at the individual level, and so improve the AIC statistic. However, this improvement need not necessarily result in a better fit to the Kaplan-Meier curve, for example if the standard parametric model adequately describes the marginal survival curve. For this, we recommend the use of the root mean-squared prediction error statistic.

## **Appendix 2**. Root mean-squared prediction error to assess marginal fit

Since the AIC statistics do not directly inform marginal goodness-of-fit (see Appendix 1) we suggest the use of a root mean-squared prediction error (RMSPE) statistic to assess the marginal fit of the different model types.

Let  $\bar{S}(t)$ be the estimated marginal survival at time $t$ from the model of interest, calculated from Equation 3, and  $\hat{S}_{KM}(t)$ be the corresponding Kaplan-Meier estimate. We define the RMSPE over all $N$ individuals in the dataset with event/censoring times $t_{i}$, $i=1,\ldots,N$, as

$RMSPE =\sqrt{\frac{1}{N}\sum_{i=1}^{N} \left( \hat{S}_{KM}\left( t_{i} \right)-\bar{S}\left( t_{i} \right) \right)^{2}}$.

## Appendix 3. Example R code: gbcsCS dataset

Example R code based on the gbcsCS dataset for calculating all-cause survival and hazard functions is presented below. The code uses the standsurv command in-built into the flexsurv package (version $\geq$2.2).

An example R script that fits Log-Normal standard parametric, excess hazard and excess hazard cure models, and obtains predictions from these models.

**NOTE: The results in this example script use US lifetables and do not match the analyses in the manuscript, which use West German lifetables downloaded from the Human Mortality Database (using the HMDHFDplus package).**

# load required packages (and install first if not already installed)
list.of.packages <- c("flexsurv", "flexsurvcure", "dplyr", "tidyr", "ggplot2",
 "gridExtra", "ggpubr", "devtools", "condSURV", "lubridate"))
new.packages <- list.of.packages[!(list.of.packages %in%
 installed.packages()[,"Package"])]
if(length(new.packages)) install.packages(new.packages)
for(pkg in list.of.packages){
 library(pkg, character.only = TRUE)
}

# 1. gbcsCS dataset ----
data(gbcsCS, package="condSURV")
## Create age at diagnosis in days - used later for matching to expected rates
gbcsCS$agedays <- floor(gbcsCS$age * 365.25)
## Survival time in years
gbcsCS$survyrs <- gbcsCS$survtime / 365.25
## Diagnosis as a date variable
gbcsCS$diag <- as.Date(as.character(gbcsCS$diagdateb), "%d-%m-%Y")
## Create sex (assume all are female)
gbcsCS$sex <- factor("female")
## 2-level grade variable
gbcsCS$grade2 <- ifelse(gbcsCS$grade==3, "3", "1/2")
## Obtain attained age and attained calendar year in (whole) years
gbcsCS <- gbcsCS %>% mutate(attained.age.yr = floor(age + survtime/365.25),
 attained.year = lubridate::year(diag + survtime))
head(gbcsCS)

## id diagdateb recdate deathdate age menopause hormone size grade nodes
## 1 1 17-08-1984 15-04-1988 16-11-1990 38 1 1 18 3 5
## 2 2 25-04-1985 15-03-1989 22-10-1990 52 1 1 20 1 1
## 3 3 11-10-1984 12-04-1988 06-10-1988 47 1 1 30 2 1
## 4 4 29-06-1984 24-11-1984 24-11-1984 40 1 1 24 1 3
## 5 5 03-07-1984 09-08-1989 09-08-1989 64 2 2 19 2 1
## 6 6 24-07-1984 08-11-1989 08-11-1989 49 2 2 56 1 3
## prog_recp estrg_recp rectime censrec survtime censdead agedays survyrs
## 1 141 105 1337 1 2282 0 13879 6.2477755
## 2 78 14 1420 1 2006 0 18993 5.4921287
## 3 422 89 1279 1 1456 1 17166 3.9863107
## 4 25 11 148 0 148 0 14610 0.4052019
## 5 19 9 1863 0 1863 0 23376 5.1006160
## 6 356 64 1933 0 1933 0 17897 5.2922656
## diag sex grade2 attained.age.yr attained.year
## 1 1984-08-17 female 3 44 1990
## 2 1985-04-25 female 1/2 57 1990
## 3 1984-10-11 female 1/2 50 1988
## 4 1984-06-29 female 1/2 40 1984
## 5 1984-07-03 female 1/2 69 1989
## 6 1984-07-24 female 1/2 54 1989

# 2. lifetables ----
# We will use the US lifetables that come with the survival package.
# First, let's reshape US lifetable to be a tidy data.frame and convert rates to
# per person-year as our survival analysis time scale will be in years
survexp.us.df <- as.data.frame.table(survexp.us, responseName = "exprate") %>%
 mutate(exprate = 365.25 * exprate)
survexp.us.df$age <- as.numeric(as.character(survexp.us.df$age))
survexp.us.df$year <- as.numeric(as.character(survexp.us.df$year))

# Now we merge in (left join) the US rates at the event times in the bc data
gbcsCS <- gbcsCS %>% left_join(survexp.us.df, by = c("attained.age.yr"="age",
 "attained.year"="year",
 "sex"="sex"))
# Create a dataset containing Grade 1/2 only
gbcsCSLowGrade <- gbcsCS %>% filter(grade2=="1/2")


# 3. Model fitting to Good prognosis group ----
# Fit a standard parametric, excess hazard and mixture-cure model to the Good
# prognosis group only.
# Demonstrate using a log-normal distribution
models <- list()
# standard parametric
models[["lnorm"]] <- flexsurvreg(Surv(survyrs, censdead)~1,
 data=gbcsCSLowGrade, dist="lnorm")
# excess hazard model
models[["lnorm.excesshazard"]] <- flexsurvreg(Surv(survyrs, censdead)~1,
 data=gbcsCSLowGrade, dist="lnorm",
 bhazard=exprate)
# excess hazard mixture-cure model
models[["lnorm.excesshazardcure"]] <- flexsurvcure(Surv(survyrs, censdead)~1,
 data=gbcsCSLowGrade,
 dist="lnorm",
 bhazard=exprate)

# 4. AIC statisics ----
# For the excess hazard models the reported AIC is from a partial likelihood
# Therefore the AIC from an excess hazard model cannot be directly compared to
# an AIC from a standard parametric model
# AIC from standard parametric model
models[["lnorm"]]$AIC

## [1] 835.449

# AIC from excess hazard model
models[["lnorm.excesshazard"]]$AIC

## [1] 802.7612

# AIC from excess hazard mixture cure model
models[["lnorm.excesshazardcure"]]$AIC

## [1] 801.0241

# 5. Predicted and extrapolated all-cause survival and hazard ----
ss.surv <- tibble()
# We use the standsurv command in the flexsurv package to make the predictions
# Predicting all-cause survival over next 30-years in increments of 0.2 years
# Standard parametric model
ss.surv.new <- standsurv(models[["lnorm"]],
 type="survival",
 t=seq(0,30, by=0.2),
 ci=FALSE
) %>%
 bind_cols(dist = "lnorm") %>%
 bind_cols("Relative Survival" = FALSE) %>%
 bind_cols(Cure = FALSE)
ss.surv <- ss.surv %>%
 bind_rows(ss.surv.new)
# Excess hazard model
# To get predictions of marginal all-cause survival, standsurv multiplies the
# predicted relative survival function with the expected survival function for
# each individual and then averages.
# We therefore must supply the expected ratetable for these calculations
# We also must scale from the ratetable time scale (days) to the regression
# model time scale (years) using the scale.ratetable argument.
ss.surv.new <- standsurv(models[["lnorm.excesshazard"]],
 type="survival",
 t=seq(0,30, by=0.2),
 ci=FALSE,
 rmap=list(sex = sex,
 year = diag,
 age = agedays
 ),
 ratetable = survexp.us,
 scale.ratetable = 365.25,
 newdata = gbcsCSLowGrade
) %>%
 bind_cols(dist = "lnorm") %>%
 bind_cols("Relative Survival" = TRUE) %>%
 bind_cols(Cure = FALSE)
ss.surv <- ss.surv %>%
 bind_rows(ss.surv.new)

## Excess hazard cure model
ss.surv.new <- standsurv(models[["lnorm.excesshazardcure"]],
 type="survival",
 t=seq(0,30, by=0.2),
 ci=FALSE,
 rmap=list(sex = sex,
 year = diag,
 age = agedays
 ),
 ratetable = survexp.us,
 scale.ratetable = 365.25,
 newdata = gbcsCSLowGrade
) %>%
 bind_cols(dist = "lnorm") %>%
 bind_cols("Relative Survival" = TRUE) %>%
 bind_cols(Cure = TRUE)
ss.surv <- ss.surv %>%
 bind_rows(ss.surv.new)
ss.surv

## # A tibble: 453 × 5
## time at1 dist `Relative Survival` Cure
## <dbl> <dbl> <chr> <lgl> <lgl>
## 1 0 1 lnorm FALSE FALSE
## 2 0.2 1.00 lnorm FALSE FALSE
## 3 0.4 0.999 lnorm FALSE FALSE
## 4 0.6 0.996 lnorm FALSE FALSE
## 5 0.8 0.992 lnorm FALSE FALSE
## 6 1 0.986 lnorm FALSE FALSE
## 7 1.2 0.978 lnorm FALSE FALSE
## 8 1.4 0.969 lnorm FALSE FALSE
## 9 1.6 0.958 lnorm FALSE FALSE
## 10 1.8 0.947 lnorm FALSE FALSE
## # … with 443 more rows

# standsurv can be re-run to predict
# all-cause hazard using type="hazard"
# excess hazard (for excess hazard models) using type="excesshazard"
# RMST using type="rmst"
# Confidence intervals can be calculated from the delta method or bootstrapping
# see help("standsurv") for more details

# 6. Plot of extrapolated survival (faceted)----
####################################################################
ss.surv <- ss.surv %>%
 mutate(Method = ifelse(`Relative Survival`==FALSE & Cure==FALSE,
 "a) Standard parametric models",
 ifelse(Cure==FALSE,
 "b) Excess hazard (no cure) models", "c) Excess hazard (cure) models")))
ss.surv$Method <- factor(ss.surv$Method,
 levels = c("a) Standard parametric models",
 "b) Excess hazard (no cure) models",
 "c) Excess hazard (cure) models"))
ggplot(ss.surv) + geom_line(aes(x=time,y=at1,color=dist)) +
 facet_wrap(~ Method, ncol=1) +
 theme_bw() +
 ylab("Survival") +
 xlab("Time (years)") +
 theme(
 strip.background = element_rect(
 color="black", fill="white", size=0, linetype="solid"
 ),
 strip.text = element_text(hjust = 0, size=10),
 legend.position="bottom",
 legend.title=element_blank()
 )


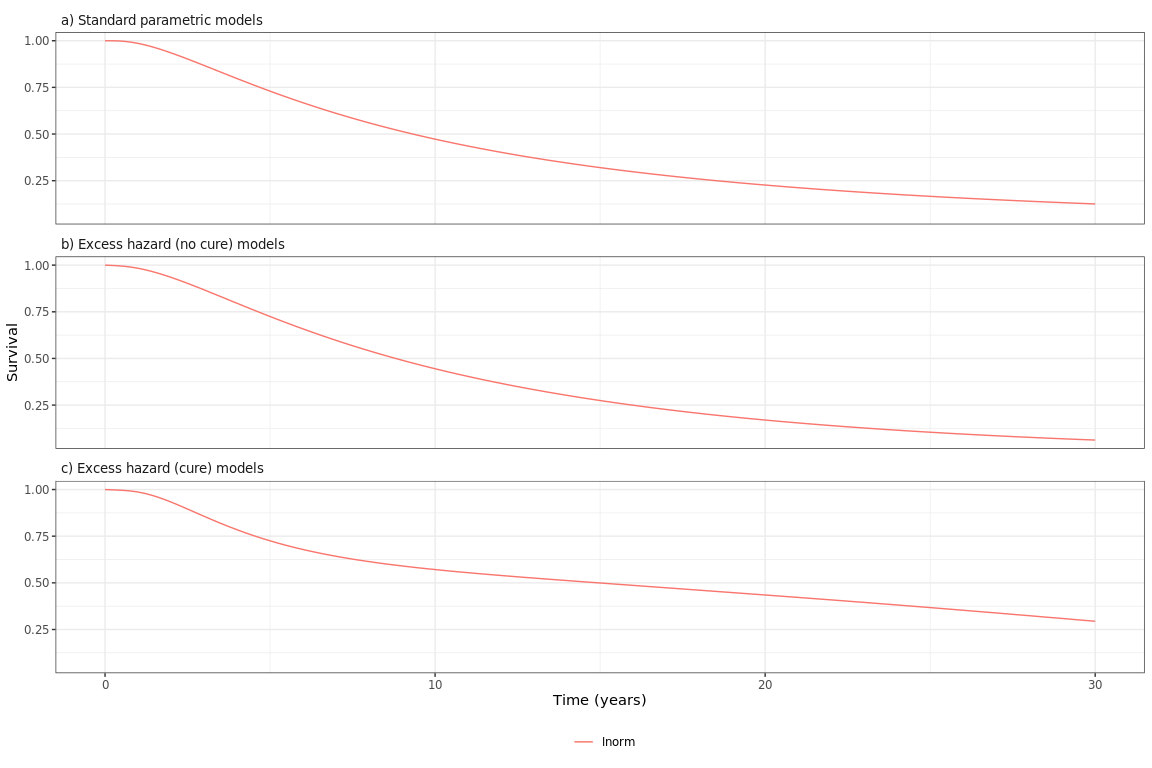


# 7. Fitting a saturated model by treatment group----
################################################################################
models.sat <- list()
# standard parametric log-normal
# both the location and ancillary parameters are a function of grade2
# this is equivalent to fitting separate log-normal models to the two groups
models.sat[["lnorm"]] <- flexsurvreg(Surv(survyrs, censdead)~grade2,
 anc=list(sdlog= ~grade2),
 data=gbcsCS, dist="lnorm")
# excess hazard model
models.sat[["lnorm.excesshazard"]] <-
 flexsurvreg(Surv(survyrs, censdead)~grade2,
 anc=list(sdlog= ~grade2),
 data=gbcsCS, dist="lnorm",
 bhazard = exprate)
# excess hazard cure model
models.sat[["lnorm.excesshazardcure"]] <-
 flexsurvcure(Surv(survyrs, censdead)~grade2,
 anc=list(meanlog= ~grade2,
 sdlog= ~grade2),
 data=gbcsCS, dist="lnorm",
 bhazard = exprate)

## 8. Predicted and extrapolated all-cause survival, hazard and hazard ratio----
################################################################################
ss.sat.surv <- tibble()
## All-cause survival
## Standard parametric (lognormal) model
## The attribute "standpred_at" gives predicted survival in long-format
ss.sat.surv.new <- attr(standsurv(models.sat[["lnorm"]],
 at=list(list(grade2="1/2"),
 list(grade2="3")),
 type="survival",
 t=seq(0,30, by=0.2),
 ci=FALSE), "standpred_at") %>%
 bind_cols(dist = "lnorm") %>%
 bind_cols("Relative Survival" = FALSE) %>%
 bind_cols(Cure = FALSE)
ss.sat.surv <- ss.sat.surv %>%
 bind_rows(ss.sat.surv.new)
## Excess hazard lognormal model
ss.sat.surv.new <- attr(standsurv(models.sat[["lnorm.excesshazard"]],
 at=list(list(grade2="1/2"),
 list(grade2="3")),
 type="survival",
 t=seq(0,30, by=0.2),
 ci=FALSE,
 rmap=list(sex = sex,
 year = diag,
 age = agedays
 ),
 ratetable = survexp.us,
 scale.ratetable = 365.25,
 newdata = gbcsCS), "standpred_at") %>%
 bind_cols(dist = "lnorm") %>%
 bind_cols("Relative Survival" = TRUE) %>%
 bind_cols(Cure = FALSE)
ss.sat.surv <- ss.sat.surv %>%
 bind_rows(ss.sat.surv.new)
## Excess hazard lognormal cure model
ss.sat.surv.new <- attr(standsurv(models.sat[["lnorm.excesshazardcure"]],
 at=list(list(grade2="1/2"),
 list(grade2="3")),
 type="survival",
 t=seq(0,30, by=0.2),
 ci=FALSE,
 rmap=list(sex = sex,
 year = diag,
 age = agedays
 ),
 ratetable = survexp.us,
 scale.ratetable = 365.25,
 newdata = gbcsCS), "standpred_at") %>%
 bind_cols(dist = "lnorm") %>%
 bind_cols("Relative Survival" = TRUE) %>%
 bind_cols(Cure = TRUE)
ss.sat.surv <- ss.sat.surv %>%
 bind_rows(ss.sat.surv.new)

## All-cause hazards and hazard ratio
ss.sat.haz <- ss.sat.HR <- tibble()
## Standard parametric lognormal
ss.sat.haz.run <- standsurv(models.sat[["lnorm"]],
 at=list(list(grade2="1/2"),
 list(grade2="3")),
 type="hazard",
 contrast = "ratio",
 atreference = 2,
 t=seq(0,30, by=0.2),
 ci=FALSE)
ss.sat.haz.new <- attr(ss.sat.haz.run, "standpred_at") %>%
 bind_cols(dist = "lnorm") %>%
 bind_cols("Relative Survival" = FALSE) %>%
 bind_cols(Cure = FALSE)
ss.sat.HR.new <- attr(ss.sat.haz.run, "standpred_contrast") %>%
 bind_cols(dist = "lnorm") %>%
 bind_cols("Relative Survival" = FALSE) %>%
 bind_cols(Cure = FALSE)
ss.sat.haz <- ss.sat.haz %>%
 bind_rows(ss.sat.haz.new)
ss.sat.HR <- ss.sat.HR %>%
 bind_rows(ss.sat.HR.new)

## Excess hazard lognormal model
ss.sat.haz.run <- standsurv(models.sat[["lnorm.excesshazard"]],
 at=list(list(grade2="1/2"),
 list(grade2="3")),
 type="hazard",
 contrast="ratio",
 atreference = 2,
 t=seq(0,30, by=0.2),
 ci=FALSE,
 rmap=list(sex = sex,
 year = diag,
 age = agedays
 ),
 ratetable = survexp.us,
 scale.ratetable = 365.25,
 newdata = gbcsCS)
ss.sat.haz.new <- attr(ss.sat.haz.run, "standpred_at") %>%
 bind_cols(dist = "lnorm") %>%
 bind_cols("Relative Survival" = TRUE) %>%
 bind_cols(Cure = FALSE)
ss.sat.HR.new <- attr(ss.sat.haz.run, "standpred_contrast") %>%
 bind_cols(dist = "lnorm") %>%
 bind_cols("Relative Survival" = TRUE) %>%
 bind_cols(Cure = FALSE)
ss.sat.haz <- ss.sat.haz %>%
 bind_rows(ss.sat.haz.new)
ss.sat.HR <- ss.sat.HR %>%
 bind_rows(ss.sat.HR.new)

## Excess hazard lognormal cure model
ss.sat.haz.run <- standsurv(models.sat[["lnorm.excesshazardcure"]],
 at=list(list(grade2="1/2"),
 list(grade2="3")),
 type="hazard",
 contrast = "ratio",
 atreference = 2,
 t=seq(0,30, by=0.2),
 ci=FALSE,
 rmap=list(sex = sex,
 year = diag,
 age = agedays
 ),
 ratetable = survexp.us,
 scale.ratetable = 365.25,
 newdata = gbcsCS)
ss.sat.haz.new <- attr(ss.sat.haz.run, "standpred_at") %>%
 bind_cols(dist = "lnorm") %>%
 bind_cols("Relative Survival" = TRUE) %>%
 bind_cols(Cure = TRUE)
ss.sat.HR.new <- attr(ss.sat.haz.run, "standpred_contrast") %>%
 bind_cols(dist = "lnorm") %>%
 bind_cols("Relative Survival" = TRUE) %>%
 bind_cols(Cure = TRUE)
ss.sat.haz <- ss.sat.haz %>%
 bind_rows(ss.sat.haz.new)
ss.sat.HR <- ss.sat.HR %>%
 bind_rows(ss.sat.HR.new)


## 9. Plot of extrapolated hazard ratios (faceted) ----
#######################################################
ss.sat.HR <- ss.sat.HR %>%
 mutate(Method = ifelse(`Relative Survival`==FALSE & Cure==FALSE,
 "a) Standard parametric models",
 ifelse(Cure==FALSE,
 "b) Excess hazard (no cure) models",
 "c) Excess hazard (cure) models")))
ss.sat.HR$Method <- factor(ss.sat.HR$Method,
 levels = c("a) Standard parametric models",
 "b) Excess hazard (no cure) models",
 "c) Excess hazard (cure) models"))
ggplot(ss.sat.HR) +
 geom_line(aes(x=time,y=ratio, color=dist)) +
 facet_wrap( ~ Method, ncol=1) +
 ylab("Hazard Ratio (Grade 1/2 vs. Grade 3)") +
 xlab("Time (years)") +
 scale_y_continuous(trans='log2',breaks=c(0.1, 0.3, 0.6, 1, 2, 4),
 lim=c(0.1,4)) +
 geom_hline(yintercept=1, alpha=0.4) +
 theme_bw() +
 theme(
 strip.background = element_rect(
 color="black", fill="white", size=0, linetype="solid"
 ),
 strip.text = element_text(hjust = 0, size=10),
 legend.position="bottom",
 legend.title=element_blank()
 )


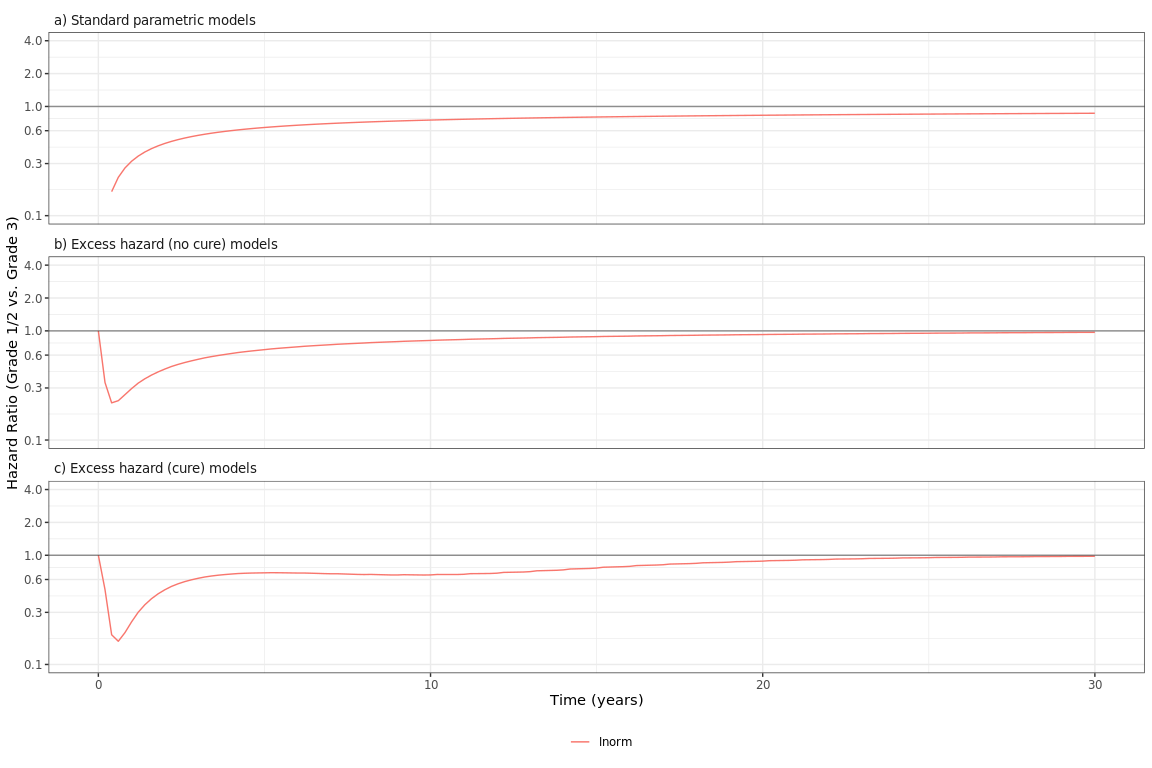

Supplement: sj-docx-1-mdm-10.1177_0272989X231184247 – Supplemental material for Survival Extrapolation Incorporating General Population Mortality Using Excess Hazard and Cure Models: A Tutorial [file sj-docx-1-mdm-10.1177_0272989X231184247.docx]
